# Analysis of Artificial Intelligence-Based Approaches Applied to Non-Invasive Imaging for Early Detection of Melanoma: A Systematic Review

**DOI:** 10.3390/cancers15194694

**Published:** 2023-09-23

**Authors:** Raj H. Patel, Emilie A. Foltz, Alexander Witkowski, Joanna Ludzik

**Affiliations:** 1 Edward Via College of Osteopathic Medicine, VCOM-Louisiana, 4408 Bon Aire Dr, Monroe, LA 71203, USA; 2 Department of Dermatology, Oregon Health & Science University, Portland, OR 97239, USAwitkowsa@ohsu.edu (A.W.); ludzik@ohsu.edu (J.L.); 3 Elson S. Floyd College of Medicine, Washington State University, Spokane, WA 99202, USA

**Keywords:** melanoma, artificial intelligence, deep learning, dermoscopy, optical coherence tomography, reflectance confocal microscopy, neural network, non-invasive imaging, in vivo imaging, early detection

## Abstract

**Simple Summary:**

Melanoma is the most dangerous type of skin cancer worldwide. Early detection of melanoma is crucial for better outcomes, but this often can be challenging. This research explores the use of artificial intelligence (AI) techniques combined with non-invasive imaging methods to improve melanoma detection. The authors aim to evaluate the current state of AI-based techniques using tools including dermoscopy, optical coherence tomography (OCT), and reflectance confocal microscopy (RCM). The findings demonstrate that several AI algorithms perform as well as or better than dermatologists in detecting melanoma, particularly in the analysis of dermoscopy images. This research highlights the potential of AI to enhance diagnostic accuracy, leading to improved patient outcomes. Further studies are needed to address limitations and ensure the reliability and effectiveness of AI-based techniques.

**Abstract:**

Background: Melanoma, the deadliest form of skin cancer, poses a significant public health challenge worldwide. Early detection is crucial for improved patient outcomes. Non-invasive skin imaging techniques allow for improved diagnostic accuracy; however, their use is often limited due to the need for skilled practitioners trained to interpret images in a standardized fashion. Recent innovations in artificial intelligence (AI)-based techniques for skin lesion image interpretation show potential for the use of AI in the early detection of melanoma. Objective: The aim of this study was to evaluate the current state of AI-based techniques used in combination with non-invasive diagnostic imaging modalities including reflectance confocal microscopy (RCM), optical coherence tomography (OCT), and dermoscopy. We also aimed to determine whether the application of AI-based techniques can lead to improved diagnostic accuracy of melanoma. Methods: A systematic search was conducted via the Medline/PubMed, Cochrane, and Embase databases for eligible publications between 2018 and 2022. Screening methods adhered to the 2020 version of the PRISMA (Preferred Reporting Items for Systematic Reviews and Meta-Analyses) guidelines. Included studies utilized AI-based algorithms for melanoma detection and directly addressed the review objectives. Results: We retrieved 40 papers amongst the three databases. All studies directly comparing the performance of AI-based techniques with dermatologists reported the superior or equivalent performance of AI-based techniques in improving the detection of melanoma. In studies directly comparing algorithm performance on dermoscopy images to dermatologists, AI-based algorithms achieved a higher ROC (>80%) in the detection of melanoma. In these comparative studies using dermoscopic images, the mean algorithm sensitivity was 83.01% and the mean algorithm specificity was 85.58%. Studies evaluating machine learning in conjunction with OCT boasted accuracy of 95%, while studies evaluating RCM reported a mean accuracy rate of 82.72%. Conclusions: Our results demonstrate the robust potential of AI-based techniques to improve diagnostic accuracy and patient outcomes through the early identification of melanoma. Further studies are needed to assess the generalizability of these AI-based techniques across different populations and skin types, improve standardization in image processing, and further compare the performance of AI-based techniques with board-certified dermatologists to evaluate clinical applicability.

## 1. Introduction

**Figure 1:**
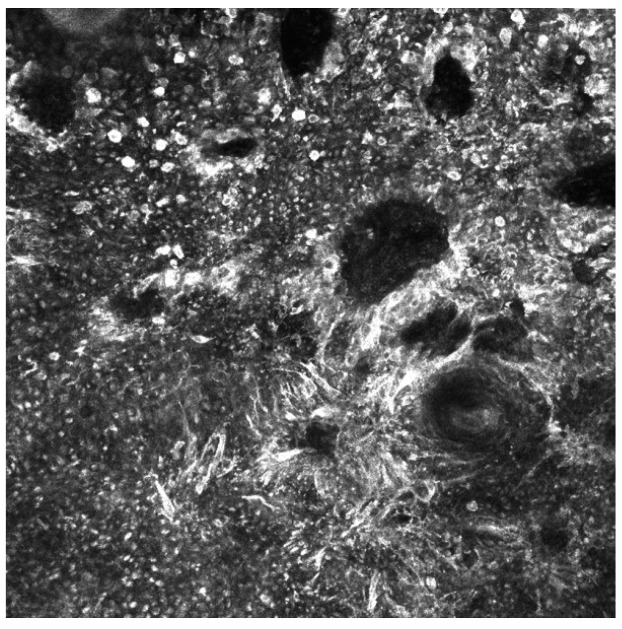
Reflectance confocal microscopy mosaic of melanoma in situ taken at the depth of the dermal–epidermal junction. Findings include an atypical ringed pattern with non-edged papillae and a non-specific pattern. There is the presence of pleomorphic atypical melanocytes (round and dendritic types) with visible dark nuclei and bright cytoplasm, as well as bundles of atypical melanocytes characteristic for melanoma. Image size: 1 mm × 1 mm.

**Figure 2:**
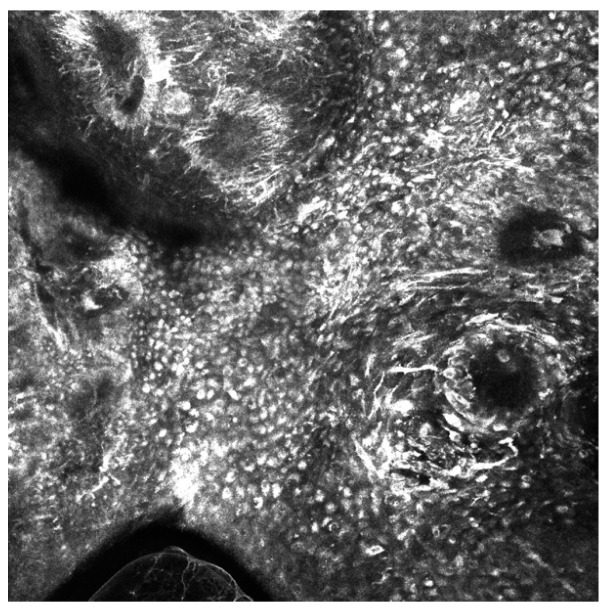
Reflectance confocal microscopy mosaic of melanoma in situ taken at depth of the epidermis. There is an irregular cobblestone pattern, the presence of folliculotropism, and pleomorphic pagetoid cells (dendritic and round types) with numerosity greater than 10 per square millimeter. Image size: 1 mm × 1 mm.

**Figure 3:**
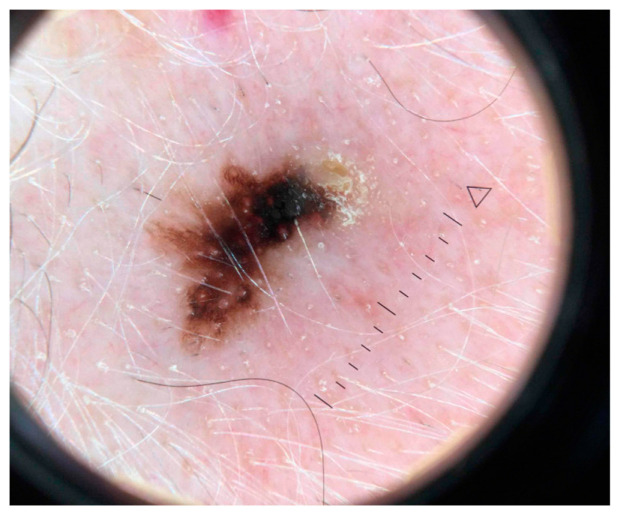
Dermoscopy of melanoma in situ demonstrating an asymmetric reticular pattern, atypical thickened network, and blue-white structures. Due to the anatomical location of the lesion (on the temple), there is the presence of perifollicular hyperpigmentation, rhomboid structures, and effacement. Scale bar in the image represents 1 cm.

It has been well established that the detection of late-stage melanoma is associated with poorer, potentially fatal outcomes. Early detection of melanoma is crucial to prevent mortality and reduce unnecessary invasive procedures including surgical biopsy [1]. The use of in vivo diagnostic imaging modalities to aid in the earlier detection of this deadly skin cancer has shown significant efficacy, particularly with techniques such as reflectance confocal microscopy (RCM), optical coherence tomography (OCT), and dermoscopy [2]. RCM uses a diode laser to provide high-resolution images in horizontal sections at the cellular level as deep as the papillary dermis, while OCT uses near-infrared light to capture microscopic images up to 2 mm below the skin’s surface (Figure 1 and Figure 2) [2,3]. Dermoscopy utilizes a dermatoscope with polarized or non-polarized light to visualize patterns and microstructures in the epidermis and superficial dermis (Figure 3) [4].

Despite the advantages of these non-invasive imaging modalities, the application of them requires substantial training and expertise, thus leading to variable results in diagnostic accuracy. Further, the quality of images may affect the interpretation and diagnostic time, calling for an unbiased and automated recognition process [5]. Artificial intelligence (AI)-based techniques have been developed to analyze images obtained by these imaging modalities, with the aim of automating the diagnostic process while providing objectivity and consistency in results. The use of AI-based techniques for the analysis of non-invasive dermatologic imaging modalities has the potential to revolutionize the way in which skin cancers are detected and diagnosed. Studies have demonstrated that machine learning algorithms have the potential to minimize the number of artifacts that need to be reviewed, speed up the diagnostic process, and reduce the number of clinic visits for patients [6]. While the use of AI in medical image recognition has grown tremendously over the past several years, its potential in the dermatological evaluation of skin cancers remains largely unrealized. High-quality and accurate algorithms rely on a balanced dataset with a high diversity of inputs to allow for precise and accurate diagnoses [7]. Thus, different studies exploring the use of machine learning-based technologies in the analysis of non-invasive dermatologic imaging modalities may show variations in results based on the differences in algorithm development and image quality. 

Furthermore, the multitude of AI-based techniques used to analyze dermatological images can complicate clinical decision-making without clear guidelines. It can be challenging to determine the feasibility of AI in clinical practice, as well as the role of machine learning, convolutional neural networks, and deep learning in these techniques. Additionally, it is important to consider the limitations of AI. While it has the potential to revolutionize the field, it can also be biased depending on the datasets and training process. For instance, datasets used to train AI models may be primarily composed of fairer skin tones, which can result in less accurate diagnoses for patients with darker skin tones [8]. Skin tones can affect the results and output based on AI model training, making it crucial to ensure that the datasets used to train these models are diverse and representative of the patient population [9]. It is also crucial to compare the diagnostic accuracy of different models trained on diverse datasets to accurately assess their potential for clinical use. An imbalanced dataset during neural network training can lead to uneven performance in the analysis of non-invasive dermatologic imaging modalities using machine learning algorithms [10,11]. Therefore, ethical considerations and guidance from the Food and Drug Administration (FDA) are necessary to ensure the safe and effective implementation of AI-based techniques in clinical settings. These considerations highlight the importance of comprehensively evaluating the current literature on the use of AI-based techniques to thoroughly understand the potential for AI in the non-invasive diagnosis of melanoma. This review aims to address these concerns and provides a better understanding of the current state of the field, with a goal of guiding the development of future research supporting the implementation of these techniques into clinical tools.

In this review, we seek to analyze and describe the current state of AI in non-invasive skin cancer detection and summarize the outcomes of various algorithms. This review provides a comprehensive analysis of the current state of AI in non-invasive skin cancer detection, highlights the clinical implications of various algorithms, and identifies areas of future research. Given the potential of these techniques to improve patient outcomes and reduce unnecessary invasive procedures, understanding the current state of the field is of paramount importance.

## 2. Materials and Methods

### 2.1. Literature Search Strategy

A literature search of PubMed/Medline, Embase, and Cochrane was used to search for papers published from 2018 to 2023, analyzing the use of AI-based techniques applied to images of malignant melanoma produced from reflectance confocal microscopy, optical coherence tomography, and dermoscopy. The PRISMA guidelines were followed during data extraction, analysis, and reporting. The following search terms were used: “melanoma”, “neural network”, “diagnosis or detection”, “carcinoma”, “lesion or growth or cancer or neoplasm or tumor or malignant or metastatic”, “computer systems”, “skin cancer detection”, “digital pathology”, “machine or deep learning”, “algorithms”, “artificial intelligence”, “skin cancers”, “diagnostic techniques and procedures”, “dermoscopy”, “reflectance confocal microscopy”, and “optical coherence tomography”.

### 2.2. Study Eligibility and Selection

**Figure 4:**
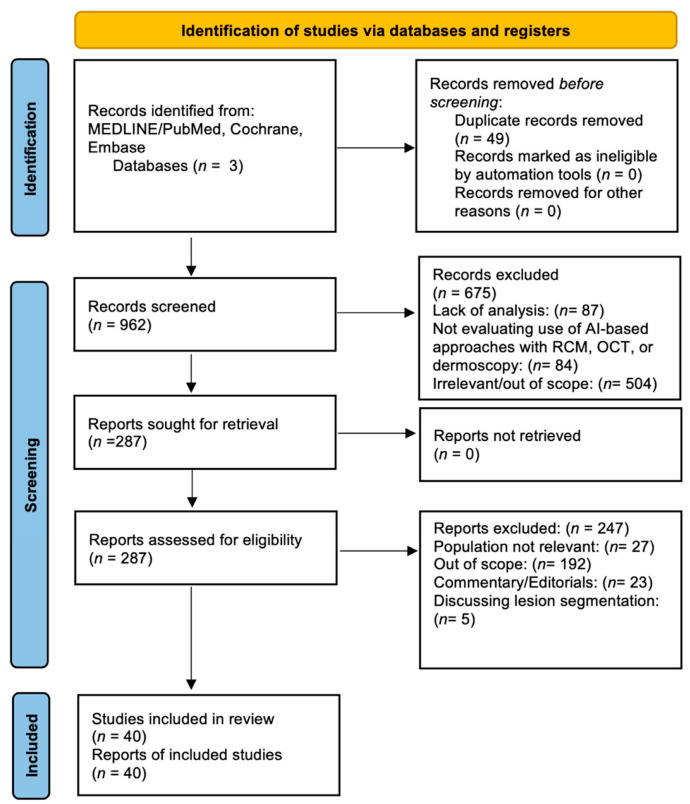
PRISMA (Preferred Reporting Items for Systematic Reviews and Meta-Analyses) 2020 Flow Diagram.

The study followed the Preferred Reporting Items for Systematic Reviews and Meta-analyses (PRISMA) guidelines, the protocol was registered in the OSF database (registration number: osf-registrations-z8tve-v1), and the search results were sorted, screened, and manually assessed by two independent authors (R.H.P. and E.F.) Any discrepancies in study selection and inclusion criteria were resolved through the input of a third reviewer (J.L.) Only original, peer-reviewed research articles in the English language were selected for review. Studies that directly compared human experts or histopathology with the AI-based evaluation of non-invasive imaging for melanoma detection (dermoscopy, reflectance confocal microscopy, and optical tomography) were part of the inclusion criteria. Studies that did not directly diagnose melanoma, did not evaluate the use of AI-based approaches with RCM, OCT, and dermoscopy, involved commentary/editorials on the subject of AI-based approaches, and discussed models for lesion segmentation without classification were excluded from the review. Finally, studies that did not evaluate diagnostic accuracy, AUC, or sensitivity/specificity rates for the early detection of melanoma using AI-based techniques were excluded (Figure 4).

From the selected articles, the following information was collected and analyzed: general information regarding the paper (authors, publication year, and origin), the purpose/objective of the work, non-invasive imaging modality, evaluation metric (diagnostic accuracy, area under the curve, sensitivity and specificity rates), and AI methodology. 

### 2.3. Study Analysis and Performance Metrics

The evaluation of the performance of AI-based approaches applied in combination with reflectance confocal microscopy, optical coherence tomography, and dermoscopy for the detection of melanoma was a critical aspect of this systematic review. The selection and extraction of appropriate performance metrics from the reviewed papers was crucial to accurately assess the diagnostic accuracy of the evaluated approaches. The three primary performance metrics chosen for this study were accuracy, sensitivity, and specificity. Accuracy is a widely used classification performance measure that indicates the percentage of accurately classified skin lesions, which is the ratio between the total number of correctly classified lesions and the overall number of examined lesions. However, accuracy may not be an appropriate metric when there is an imbalance in the distribution of target classes in the dataset. Therefore, sensitivity and specificity, which are unaffected by class imbalance, were also considered in this study. Sensitivity is a measure of the proportion of true positive cases, i.e., the number of cases correctly identified as melanoma, in relation to the total number of actual melanoma cases in the dataset. Specificity, on the other hand, measures the proportion of true negative cases, i.e., the number of cases correctly identified as non-melanoma, in relation to the total number of actual non-melanoma cases in the dataset. The mean sensitivity and specificity of papers comparing AI-based techniques with dermatologist performance was calculated in this study. For studies evaluating OCT and RCM, accuracy rates were studied due to the mutual availability of this metric in the reviewed papers. 

Other performance metrics, such as the area under the curve (AUC), positive predictive value (PPV), and negative predictive value (NPV), were also reported and discussed, when available. AUC is a commonly used performance metric that provides a comprehensive evaluation of the performance of a classification model across all possible decision thresholds. PPV and NPV are metrics that indicate the proportion of positive and negative cases, respectively, that are correctly identified by the model. The performance metrics were used to analyze and compare the diagnostic accuracy of the evaluated AI-based approaches. The analysis of these metrics allowed for a comprehensive evaluation of the effectiveness of AI-based approaches in the detection of melanoma, as well as the identification of potential areas for improvement and future research.

## 3. Results

After initial screening, a total of 287 articles were assessed for eligibility. The PRISMA flow diagram summarizing the identification of studies is shown in Figure 1. Following a careful evaluation of their titles and abstracts, 40 articles were ultimately included in this systematic review. Most of the articles applied AI-based approaches to dermoscopic images (n = 37), followed by optical coherence tomography images (n = 1) and reflectance confocal microscopy images (n = 2) (Table 1, Table 2 and Table 3). 

### 3.1. Artificial Intelligence-Based Approaches Applied to Dermoscopic Images

Deep learning has gained significant attention in the field of dermatology, particularly in the analysis of dermoscopy images. Dermoscopic images of melanoma have been widely used in the development and evaluation of deep learning-based models. The majority of articles in our review trained their algorithms on publicly available datasets of dermoscopic images of melanoma that were confirmed by either histopathology, follow-up examination, expert consensus, or confirmation by in vivo confocal microscopy. A summary of these publicly available datasets is shown in Table 4.

Foahom Gouabou et al. presented a deep learning ensemble method to obtain the accurate computer-assisted diagnosis (CAD) of melanoma [12]. This was undertaken through the evaluation of 1113 dermoscopic images of melanoma lesions extracted from a public dataset. The proposed framework achieved an area under the receiver operating curve (AUROC) of 0.93 for melanoma detection and outperformed similar existing methods within this task. Furthermore, this study also evaluated the decision-making process between the algorithm versus a trained dermatologist in distinguishing between benign keratosis and melanoma. The algorithm demonstrated enhanced efficiency and accuracy in diagnosing challenging-to-classify pigmented lesions for the task (*p* = 0.90 for melanoma), while also offering a transparent and unbiased decision-making process. Marchetti et al. presented one of the earliest studies, in 2016, comparing the diagnostic accuracy of dermoscopic melanoma images using deep learning algorithms with dermatologists [13]. This study uniquely used five individual methods to combine individual automated predictions into “fusion” algorithms. Notably, the top fusion model outperformed eight experienced dermatologists. The average sensitivity and specificity of dermatologists in accurate classification (benign lesions vs. malignant) was 82% and 59%, respectively. The study found that the best fusion computer algorithm achieved a remarkable ROC area of 0.86, demonstrating a significant improvement over the mean ROC area of 0.71 observed among the eight readers in classification (*p* = 0.001).

Subsequently, in 2020, Marchetti et al. proposed a similar study in which the best performing algorithm considerably outperformed eight dermatologists and nine dermatology trainees (*p* < 0.001) [14]. Xia et al. proposed a two-stage approach that identifies all lesions in an image, estimates their likelihood of malignancy, and generates an image-level likelihood for high-level screening [15]. This strategy led to an AUC of 0.959 based on dermoscopic images, which was augmented to an AUC of 0.961 using a publicly available dataset from ISIC 2018. This two-stage model demonstrated satisfactory performance and could be used in a PCP triage for dermatology at scale for images concerning malignancy as a complete end-to-end system. Xin et al. proposed a novel transformer network, SkinTrans, which demonstrated 94.1% accuracy on a clinical dataset of 1113 melanoma dermoscopic images, performing better than traditional CNN models [16]. 

In a larger study utilizing several publicly available datasets, Singh et al. applied a segmentation model to four public datasets, PH2, ISIC 2017, ISIC 2018, and ISIC 2019, yielding an accuracy score of 99.50%, 99.33%, 98.56%, and 98.04%, respectively [17]. The proposed method had a significantly higher score in sensitivity and specificity in the field of the diagnosis of melanoma lesions. In comparison, Kaur et al. proposed an automated melanoma classifier based on a deep convolutional neural network to accurately classify benign vs. malignant melanoma. Images were obtained from the ISIC 2016, ISIC 2017, and ISIC 2020 datasets, for which the proposed DCNN classifier achieved accuracy rates of 81.41%, 88.23%, and 90.42%, respectively [24]. This model demonstrated favorable performance in comparison with other neural networks, while offering an advanced framework for the automation of the diagnostic process. 

Naeem et al. proposed a deep learning-based framework for the multiclassification of skin cancer using dermoscopic images [18]. For melanoma, the training set included 3166 images with a validation set of 452 images and a testing set of 904 images. This system achieved better performance as compared to four pre-trained classifiers, with accuracy of 92.18% for the classification of melanoma. It achieved an AUC of 0.9833, recall of 99.9%, precision of 92.21%, an f1-score of 91.37%, and accuracy of 92.21%. Lee et al. introduced a suite of deep neural network designs tailored to melanoma detection from dermoscopy images [19]. This study produced sensitivity of 92.8%, a PPV of 78.5%, and an NPV of 91.2%. This model allows for the use of a pre-screening tool in the diagnostic process of melanoma while providing a fine balance between computational efficiency and accuracy. Fraiwan et al. utilized the HAM1000 dataset of dermoscopic images to classify melanoma vs. non-melanoma skin cancers [20]. The melanoma class was detected with 71% sensitivity (i.e., recall) but 43.1% precision. The highest reported accuracy rate from this study was 76.7%. 

Further, Martin-Gonzalez et al. developed a deep learning-based tool to differentiate between benign skin lesions and melanoma in the hospital setting [22]. This was based on a dataset of 232 dermoscopic images, which were analyzed by the system. The nevus group had a significantly lower mean diagnostic threshold (27.12 ± 35.44%) compared to the melanoma group (72.50 ± 34.03%), with a *p*-value of less than 0.001. The area under the ROC curve was 0.813. Sensitivity of 0.691, specificity of 0.802, and accuracy of 0.776 were achieved at a diagnostic threshold of 67.33%. Of note, this study did not incorporate the use of public databases. Lu et al. proposed a deep learning-based classification system for melanoma detection using dermoscopic images from the HAM10000 dataset [23]. This method offered an accuracy rate of 100% for the detection of melanoma, a sensitivity rate of 94.05%, and a precision rate of 97.07%. Vaiyapuri et al. developed a novel computational intelligence-based melanoma detection and classification approach using dermoscopic images with maximum accuracy of 97.50% [21]. Arshad et al. similarly used the HAM10000 database to apply an automated framework for multiclass skin lesion classification, obtaining an accuracy rate of 91.7% [25]. 

Xing et al. proposed a novel Zoom-in Attention and Metadata Embedding (ZooME) melanoma detection network and applied this to the ISIC 2020 dataset composed of 33,126 dermoscopy images [26]. This model achieved a 92.23% AUC score, 84.59% accuracy, 85.95% sensitivity, and 84.63% specificity. Thus, this model uniquely demonstrates a benefit in the extraction and utilization of unique pathological information from dermatoscopic images and in embedding various patient demographics for better prediction. Pham et al. introduced a novel technique that enhances melanoma prediction on an imbalanced dataset through a reconstructed CNN architecture and optimized algorithms [27]. The training dataset, consisting of 17,302 melanoma and nevus images, is the largest dataset utilized in this context. A comprehensive comparison was conducted between the model’s performance and that of 157 dermatologists from 12 university hospitals in Germany, all based on the same dataset. The findings revealed that the proposed approach outperformed all 157 dermatologists and demonstrated superior performance to the state-of-the-art method, achieving an impressive area under the curve of 94.4%, sensitivity of 85.0%, and specificity of 95.0%.

Another deep learning-based approach, proposed by Nawaz et al., evaluated on three standard datasets (ISBI 2016, ISIC 2017, and PH2), demonstrated that the presented method outperformed current approaches [29]. This method achieved average accuracy of 95.40, 93.1, and 95.6% on the ISIC 2016, ISIC 2017, and PH2 datasets, respectively, highlighting its robustness in skin lesion recognition and segmentation. This approach combines faster region-based convolutional neural networks (RCNN) with fuzzy k-means clustering (FKM), allowing for considerable improvements in melanoma detection even in the presence of image artifacts such as hair, blood vessels, lighting variations, and noise. In their work, Kim et al. introduced a publicly available deep learning algorithm for the classification of malignant melanoma. The proposed approach leveraged skin lesion images and expert labeling outcomes obtained from convolutional neural networks [28]. The U-Net model employed achieved a notable Dice similarity coefficient of 81.1% when compared to the expert labeling results. Moreover, the algorithm demonstrated high classification accuracy of 80.06% for malignant melanoma cases. These findings highlight the effectiveness of the proposed approach in accurately identifying and classifying malignant melanoma.

To overcome the problem of class imbalance in datasets, Sayed et al. proposed a new model for the classification of skin lesions using ISIC 2020 [30]. This study utilized a hybrid version of a convolutional neural network architecture and bald eagle search (BES) optimization to solve this issue. This deep network model for the prediction of melanoma skin cancer used fewer parameters and achieved overall accuracy of 98.37%, with specificity of 96.47%, sensitivity of 100%, an f-score of 98.40%, and an area under the curve of 99%. These findings demonstrated that the proposed model was both robust and efficient, outperforming VGG19, GoogleNet, and ResNet50.

Moreover, Foahom-Gouabou et al. proposed an ensemble of convolutional neural networks (CNNs) with a directed acyclic graph to aggregate binary CNNs, resulting in the best balanced accuracy (76.6%) among multiclass CNNs and other related works on the ISIC 2018 public dataset [31]. This method is noteworthy for its hierarchical workflow, which promotes transparency in the decision-making process and, as a result, simplifies the interpretation for dermatologists. Alsaade et al. aimed to develop a system for the diagnosis of skin cancer using deep learning and traditional AI machine learning algorithms [32]. The system was evaluated on dermoscopy images from two datasets, PH2 and ISIC 2018. The proposed method outperformed state-of-the-art methods for both datasets, with the artificial neural network (ANN) model achieving the highest accuracy of 97.50% for PH2 and 98.35% for ISIC 2018 compared to the convolutional neural network (CNN) model. 

Two papers developed similar models based on the ISIC 2017 dataset. Iqbal et al. developed a deep learning model for the automated multiclass classification of skin lesions using dermoscopic images from ISIC databases [33]. The proposed deep convolutional neural network (DCNN) approach outperformed state-of-the-art algorithms, achieving 94% precision, 93% sensitivity, and 91% specificity in ISIC-2017 with a 0.964 AUROC. This proposed approach provides a feasible way to automate and expedite the skin lesion classification task, potentially saving effort, time, and human lives. Similarly, Acosta et al. developed a deep learning-based approach that involves a two-stage process using mask and region-based convolutional neural networks (CNNs) and a ResNet152 structure for lesion classification [34]. The model achieved an accuracy and balanced accuracy increase of 3.66% and 9.96%, respectively, compared to the best results reported in the 2017 International Symposium on Biomedical Imaging Challenge dataset. This model boasts an excellent balance between overall accuracy (0.904), sensitivity (0.820), and specificity (0.925). 

In 2017, Tognetti et al. aimed to develop a deep convolutional neural network, called iDCNN_aMSL, to support dermatologists in the differentiation of early melanoma from atypical nevi using dermoscopic images and clinical data [35]. The model was compared to the intuitive diagnoses of dermatologists with different experience levels and achieved the best accuracy, reducing the ratio of inappropriate excision. This model achieved an area under the curve of 90.3%, sensitivity of 86.5%, and specificity of 73.6% compared to the intuitive diagnoses of dermatologists (sensitivity of 77% and specificity of 61.4%), and it can provide valuable assistance to dermatologists in making informed medical decisions that can help to reduce the number of unnecessary excisions. Guo et al. proposed a deep convolutional neural network trained with both cross-entropy and covariance discriminant loss [37]. This approach improves the model outputs and extracted features simultaneously, and a new embedding loss is designed to separate the features of melanoma and non-melanoma images more effectively. The proposed method achieved sensitivity of 0.942 and 0.917 on the ISBI 2018 Skin Lesion Analysis dataset, demonstrating its efficacy in melanoma recognition.

R K et al. also presented a deep convolutional neural network for automated melanoma detection that can adapt to different hardware and software limitations [38]. The network was trained on dermoscopic skin images from open sources and achieved high average values for accuracy, sensitivity, and specificity of 82.95%, 82.99%, and 83.89%, respectively, when tested on a dataset of 2150 images. Minagawa et al. compared the performance of 30 Japanese dermatologists with a deep neural network (DNN) for the dermoscopic diagnosis of skin tumors in different datasets [39]. Interestingly, the study found that the dermatologists’ sensitivity for malignancy prediction was significantly higher for the Shinshu dataset (Japanese only) compared to the ISIC dataset (predominately fair-skinned), and the DNN had higher specificity than the human readers. The study suggests that a DNN may help to improve the diagnostic performance for skin tumors in patients with different skin types and may be more effective at identifying malignant features after initial screening by dermatologists.

Further, Nasiri et al. presented an approach for the early detection of melanoma using deep learning in a case-based reasoning (CBR) system [40]. The system, called DePicT Melanoma Deep-CLASS, utilizes a convolutional neural network (CNN) composed of sixteen layers to classify skin lesions as benign or malignant melanoma. The efficiency of this system was demonstrated using the ISIC Archive dataset and the proposed method was shown to be effective in malignancy detection, with validation on 1796 dermoscopy images. Winkler et al. aimed to investigate the diagnostic performance of a deep learning convolutional neural network (CNN) for melanoma diagnosis across different melanoma localizations and subtypes [41]. Each set included 30 melanomas and 100 benign lesions of related localizations and morphology. The study found that the CNN showed high-level performance in superficial spreading melanomas, nodular melanomas, and lentigo maligna melanomas and facial solar lentigines/seborrhoeic keratoses/nevi. In acrolentiginous melanomas, the CNN’s sensitivity was lower, but high specificity was noted. 

Phillips et al. conducted a study with the aim of assessing the accuracy of a neural network called Deep Ensemble for Recognition of Melanoma (DERM) in detecting malignant melanoma from dermoscopic images of pigmented skin lesions [42]. The DERM model was trained and tested using a dataset consisting of 7102 dermoscopic images encompassing both melanoma and benign lesions. The findings revealed that DERM achieved an impressive area under the ROC curve (AUC) of 0.93, along with sensitivity of 85.0% and specificity of 85.3%. Additionally, the study conducted a comprehensive meta-analysis that examined the accuracy of naked-eye examination, with or without dermoscopy, performed by specialist and general physicians. The meta-analysis demonstrated that primary care physicians achieved an AUC of 0.83, with sensitivity of 79.9% and specificity of 70.9%, while dermatologists achieved an AUC of 0.91, with sensitivity of 87.5% and specificity of 81.4%. The study suggests that DERM has the potential to be used as a decision support tool in primary care by providing dermatologist-grade recommendations on melanoma likelihood. Following their previous work, the same research group conducted a subsequent study to evaluate the accuracy of an artificial intelligence algorithm in detecting melanoma in dermoscopic images of skin lesions captured using smartphones and digital single-lens reflex (DSLR) cameras [51]. The algorithm achieved an impressive area under the receiver operator characteristic (ROC) curve of 91.8% when compared to histopathological diagnosis. Notably, at 100% sensitivity, the algorithm achieved specificity of 64.8%, while clinicians achieved specificity of 69.9%. 

Several studies have gone beyond the traditional binary perspective, presented by Brinker et al., Haenssle et al., and Marchetti et al., in an effort to undertake multiclass classification tasks [43,44,46]. In 2019, Brinker et al. compared the diagnostic accuracy of a deep convolutional neural network (CNN) with that of dermatologists in the classification of melanoma and nevi images [43]. A total of 804 dermoscopic images were presented to nine dermatologists, and the CNN achieved higher sensitivity and specificity in lesion classification than the dermatologists. The study concluded that automated dermoscopic melanoma image classification was significantly superior to both junior and board-certified dermatologists. Subsequently, Brinker et al. trained another deep learning algorithm on open-source images, which outperformed 136 of 157 dermatologists in classifying dermoscopic melanoma images [44]. The algorithm was measured in terms of sensitivity, specificity, and receiver operating characteristics and achieved higher scores than dermatologists of all levels of experience. 

Similarly, in a 2020 study by Marchetti et al., computer algorithms from an international melanoma detection challenge outperformed eight dermatologists and nine dermatology residents in diagnosing melanoma from dermoscopy images, with an area under the receiver operating characteristic curve of 0.87 compared to 0.74 and 0.66, respectively [14]. The algorithm had superior specificity at the dermatologists’ overall sensitivity, and the imputation of algorithm classifications increased the dermatologists’ sensitivity and specificity. Although the study’s artificial setting lacked the full spectrum of skin lesions and clinical metadata, the results suggest that deep neural networks have the potential to improve human performance in skin lesion classification.

In one of the earliest studies that uniquely provided additional clinical information to dermatologists within the reader study, Haenssle et al. compared the diagnostic performance of a deep learning convolutional neural network (CNN) for dermoscopic melanoma recognition to that of 58 dermatologists, including 30 experts [46]. The CNN had higher specificity than the dermatologists at their respective sensitivity and achieved a greater area under the curve (AUC) for receiver operating characteristics (ROC) than the dermatologists. The study suggests that dermatologists may benefit from assistance by a CNN’s image classification.

Hagerty et al. proposed a unique fusion approach of conventional image processing and deep learning to diagnose melanoma dermoscopy images [45]. The fusion technique combines three handcrafted, biologically inspired image processing modules and one clinical information module with a ResNet50 deep learning network. Using logistic regression to ensemble the scores from both processing arms, the fusion technique achieved classification accuracy of 0.94, compared to 0.87 for the deep learning classifier alone and 0.90 for the conventional image processing classifier alone. The study suggests the further investigation and development of fusion techniques for melanoma diagnosis. Li et al. proposed two separate deep learning methods to address the challenges in the accurate recognition of melanoma in dermoscopy images [47]. The methods included lesion segmentation, lesion dermoscopic feature extraction, and lesion classification tasks. The proposed deep learning frameworks achieved promising accuracy of 0.753 for task 1, 0.848 for task 2, and 0.912 for task 3 on the ISIC 2017 dataset. This study suggests that the reliable automatic detection of skin tumors using deep learning networks can increase the accuracy and efficiency of pathologists.

In contrast with the majority of studies in this section, Gareau et al. developed a transparent machine learning technology to differentiate melanoma from nevi in dermoscopy images, and an interface for sensory cue integration [36]. The interpretable machine learning algorithm outperformed the leading deep learning approach 75% of the time. In lieu of using a deep learning-based approach, this study aimed to provide a transparent approach that results in greater medical accountability and confidence than a CNN.

### 3.2. Artificial Intelligence-Based Approaches to Analysis of Reflectance Confocal Microscopy (RCM) Images

Reflectance confocal microscopy (RCM) has emerged as a valuable tool in dermatology for the diagnosis of skin tumors, including melanoma and non-melanoma skin cancers, as well as for the interpretation and management of inflammatory and infectious skin diseases [52]. This technique allows for the real-time in vivo visualization of the epidermis to the papillary dermis, with comparable resolution to histology [53]. By allowing the dermatologist to essentially perform a “virtual biopsy” of skin lesions, it can aid in reducing unnecessary invasive procedures and skin biopsies [54]. RCM can increase the specificity and sensitivity of diagnosis in most skin cancers, guide biopsy in suspicious lesions, and aid in mapping the margins of large tumors for proper surgical excision [52]. It can also be used to follow the clinical and therapeutic evolution of skin diseases. 

Despite its benefits, the subjectivity of this method and the reliance on user interpretation create the potential for variations in diagnosis [52]. This opens up an opportunity for AI in allowing for the better delineation of the dermal–epidermal junction in RCM images, while also expediting the diagnostic process and aiding the novice, untrained user. By providing an objective analysis, AI applied to RCM images can allow for a reduction in human error, improvement in diagnostic accuracy, and enhancement in the efficiency and reliability of diagnoses. In 2019, Wodzinski et al. proposed a convolutional neural network approach to classify skin lesions using reflectance confocal microscopy (RCM) mosaics [49]. The dataset consisted of 429 RCM mosaics and was divided into three classes: melanoma, basal cell carcinoma, and benign naevi. The test set classification achieved higher accuracy as compared to medical, confocal users. This classification system provides a useful tool for the early, non-invasive detection of melanoma. In 2021, D’ Alonzo presented a weakly supervised machine learning model for the segmentation of RCM mosaics into “benign” and “aspecific” regions [50]. By using a deep neural network, the trained model achieved an average area under the curve of 0.969 and a Dice coefficient of 0.778, showing the feasibility of the spatial localization of aspecific regions in RCM images, making the diagnostics decision model more interpretable to clinicians. 

### 3.3. Artificial Intelligence-Based Approaches Applied to Optical Coherence Tomography (OCT) Images

Optical coherence tomography (OCT) is an interferometric imaging method, originally used for optical imaging, that has since been translated to cutaneous evaluations. This imaging method provides real-time views of the superficial layers of the skin [55]. It uses an infrared broadband light source to provide a view into the skin up to a depth of 1–2 mm, with a resolution between 15 and 3 μm depending on the system used [55]. OCT can be used to evaluate non-melanoma skin cancers, inflammatory diseases, and parasitic infestations, as well as for the investigation of nails [56]. OCT provides a quick and useful diagnostic imaging technique that can be used alone or in combination with other non-invasive imaging tools like dermoscopy, high-frequency ultrasound, and confocal laser scan microscopy [57]. 

While OCT has been utilized as an imaging modality for the diagnosis of basal cell carcinoma and other keratinocyte carcinomas, its use in the diagnosis of malignant melanoma remains under investigation. Initial studies have shown that OCT can detect the diagnostic features of melanoma, such as epidermal psoriasiform hyperplasia, melanocytic nests, and vertical icicle-shaped structures, and may be useful in the preoperative risk stratification of patients with melanoma [58]. However, while OCT shows promise in the diagnosis of malignant melanoma, its sensitivity and specificity as a standalone diagnostic tool are not yet convincing [59]. Angiographic OCT shows potential in the diagnosis and staging of melanoma, as it can detect early changes in vessel morphology from dysplastic nevi to melanoma [60]. Although traditional OCT has not yet been applied to AI-assisted diagnosis for melanoma, a similar protocol using vibrational OCT was described in one paper in our systematic search. While traditional OCT uses near-infrared light to create detailed, high-resolution images of biological tissues, vibrational OCT combines traditional OCT with a mechanical vibration stimulus to measure the vibrational properties of tissue structures, providing additional information about tissue composition and function. This technique can be used to image and differentiate between certain tissue features, such as the presence of collagen or elastin. This added feature can be useful in distinguishing melanoma from benign moles. Thus, while traditional OCT provides high-resolution images and an evaluation of the thickness and structure of melanoma lesions, vibrational OCT may offer additional information that can aid in the differentiation between melanoma and benign nevi. 

In 2022, Silver et al. explored the use of vibrational optical coherence tomography (VOCT) along with machine learning to differentiate between normal skin and different skin cancers non-invasively [48]. The results demonstrated that machine learning, along with the height and location of the VOCT mechanovibrational peaks, can be used to differentiate between normal skin and different cancerous lesions, including melanoma, with sensitivity and specificity rates of 83.3% and 77.8% [48].

## 4. Discussion

To the best of our knowledge, this systematic review obtained the largest number of recent studies (n = 40) published regarding the application of AI-based models to the non-invasive imaging of melanoma. The vast majority of these studies applied a deep learning-based model on dermoscopic images, as research involving AI-based OCT and RCM for the early detection of melanoma continues to develop. These non-invasive modalities differ significantly in the diagnostic information that they offer and the technical requirements for their handling, including the type of features, image size, and necessary preprocessing. Current state-of-the-art work in the field of AI-based melanoma detection primarily involves the application of deep learning models to dermoscopic images. The unique contribution of our review lies in its comprehensive analysis of recent studies and its emphasis on the potential of AI for early melanoma detection using three of the major non-invasive imaging modalities.

The main question addressed by our review is whether the application of AI-based models to the non-invasive imaging of melanoma is feasible, efficient, and beneficial compared to modern day standards, and whether this application provides for the early detection of melanoma. Consequently, this systematic review sought to address this gap by emphasizing the potential of deep learning techniques for early melanoma detection and the importance of further research efforts in this area. Since the development of these deep learning models and the datasets used for their training differ, a direct comparison between them was impractical. Hence, our discussion focused on the clinical utility of these results and established the groundwork for future studies of AI development for the evaluation of melanoma. 

### 4.1. Clinical Utility and Perceptions of AI in Dermatology

Among the 40 studies included within this systematic review, all showed robust performance in melanoma identification through AI-based algorithms. The majority of the studies were based on dermoscopic image sets as OCT and RCM datasets have not yet been widely applied to AI due to the lack of many public datasets for these categories. Studies within the dermoscopic image category primarily utilized deep learning; however, some papers applied a fusion method or conventional machine learning to datasets [13,45]. In studies directly comparing algorithm performance on dermoscopy images to dermatologists, AI-based algorithms achieved a higher ROC (>80%) in the detection of melanoma [27,39,43,44,46]. In these comparative studies using dermoscopic images, the mean algorithm sensitivity was 83.01% and the mean algorithm specificity was 85.58%. Thus, these comparative studies indicate the great potential that AI holds in diagnosis based on dermoscopic images of melanoma. While AI-based clinical OCT has shown promising results in the diagnosis of keratinocyte carcinomas, its use in the diagnosis of malignant melanoma is still in its infancy. AI-based algorithms have not yet been applied to traditional OCT; however, the potential of using vibrational optical coherence tomography (VOCT) with machine learning to improve the accuracy of melanoma diagnosis remains promising [48]. Studies evaluating the use of AI on RCM images of melanoma have also shown promising results in improving the accuracy and efficiency of diagnosis and allowing for the potential reduction of unnecessary biopsies [49,50]. AI also has the potential to increase the accuracy and reproducibility of results. These advances can have significant implications for patients, as well as clinicians, by providing earlier, non-invasive detection of melanoma and other skin cancers, leading to better outcomes and reduced healthcare costs [61]. 

The use of AI within the medical field continues to rapidly grow. Within the field of dermatology, where the early diagnosis and treatment of melanoma is key, AI can be greatly beneficial in addressing various diagnostic barriers. In one study seeking to explore how patients view AI and perceive its use in skin cancer screening, patients appeared to be receptive to the use of AI for skin cancer screening under the condition that the physician–patient relationship was preserved [62]. Given the need for heterogenous clinical and photographic data to expand the potential for AI, understanding patients’ perceptions of this tool is essential. 

In our review, most papers reviewing dermoscopic images for AI-assisted diagnosis of melanoma utilized the International Skin Imaging Collaboration (ISIC) publicly available dataset. Although this robust dataset has a large number of dermoscopic images, it mainly encompasses data on lesions from light-skinned patients in the western world (United States, Europe, and Australia), with reduced representation of skin lesions from Asian or other darker-skinned populations [11]. Thus, the future development of public datasets or clinical collection of dermoscopic images should focus on expanding datasets to include images from all skin tones in order to develop more powerful algorithms that can be reliably applied to all ethnicities. Achieving diverse datasets that are representative of skin lesions seen in clinical practice across the world can also help to realize the full potential of AI in countries that may not have easy access to dermatological care. 

The future integration of AI into clinical platforms can aid in reaching populations such as rural communities, which already suffer from disparities in melanoma incidence and higher mortality in certain parts of the U.S. [63,64]. By combining these techniques with dermoscopy and visual inspection, the diagnosis of melanoma can be made more efficient and accessible in areas where dermatologists are not readily available.

### 4.2. Ethical Implications

The integration of AI in dermatology necessitates the consideration of ethical, legal, and patient privacy issues [65]. Although the use of AI could potentially be helpful in various areas of a traditional dermatology practice, ranging from triaging patients to allowing for the use of non-invasive diagnosis or aiding in the early detection of skin cancer, its potential flaws must be recognized and taken into account. As many AI models have been trained mainly on European or East Asian populations, the relative lack of training on darker skin pigmentation may limit the overall diagnostic accuracy [8]. Imbalanced datasets may thus be susceptible to outputting incorrect results and could result in theoretical consequences for both the physician and patient if treatment or skin surgery is undertaken without a supplemental diagnosis [27]. On the contrary, AI-based algorithms may also be susceptible to false negatives, as demonstrated through our review, where the sensitivity and specificity are not 100%. Thus, it may be important to notify and educate patients about AI and its use in their diagnosis if undertaken. The potential flaws of AI also emphasize its role in serving as a supplement or diagnostic aid in the clinic, and not as a replacement for board-certified dermatologists. As AI continues to grow beyond medicine, it may be necessary to launch future public health educational campaigns to educate patients about its use and impact on their medical care.

### 4.3. Limitations

As many of the papers evaluated AI-based algorithms on different datasets, a direct comparison of the efficacy of the various methods was not feasible. Further, a meta-analysis of these studies was not plausible due to the variability in performance metrics among studies. The QUADAS-2 tool, which is typically used to analyze diagnostic accuracy studies, could not be applied due to incomplete information about certain domains in the reviewed papers. Additionally, while deep learning was the primary algorithm used, differences in image processing and reporting standards can make it difficult to draw definitive conclusions. The consideration of methodological improvements for future studies should include establishing standardized reporting and evaluation methods and considering diverse datasets. Various factors can enhance the reproducibility for future datasets. This includes the sharing of code, models, and image datasets. The challenges of new and emerging data can be addressed by continuously updating and expanding the datasets used for training and validation, including diverse images representing various skin types and populations.

Moreover, the generalizability of these algorithms poses an important issue. The majority of studies focused on patients with fairer skin types; thus, the translation of the studies’ findings to darker skin or patients with other skin variations may be a challenge. To overcome this issue, future studies should aim to include diverse datasets representing a wide range of skin tones and ethnicities. Artifacts such as the presence of hair in the image or skin texture differences can impact the accuracy of AI-based algorithms and need to be considered when implementing them in clinical practice. Image quality is another important factor to consider when implementing AI-based algorithms in clinical practice. Differences in image acquisition and quality, such as zoom level, focus, lighting, or surgical ink markings, can introduce additional bias and impact the accuracy of AI-based algorithms. These issues must be addressed and overcome to realize the full potential of AI-based techniques for the early detection of melanoma. By expanding the diversity of datasets, the power and reliability of AI-based algorithms can be improved, ensuring their applicability across different populations and clinical settings.

It is also important to note that our systematic review may be subject to publication bias. Studies that report favorable results for AI-based algorithms are more likely to be published, while studies with lower accuracy rates or higher false positives or negatives may be less likely to be reported. Lastly, studies directly comparing AI-based algorithms to dermatologists or comparing OCT and RCM with AI application were found to be limited due to the early stages of these techniques. In summary, while our review highlights the potential of AI in dermatology, it is important to recognize and address these limitations before implementing AI-based algorithms in clinical practice. Further research is needed to better understand the risks of potential biases and limitations of these techniques and to develop standardized reporting and evaluation methods for consistent and reliable results.

## 5. Conclusions

Our systematic review of 40 recent studies highlights the great potential of AI-based models for the non-invasive imaging of melanoma. The vast majority of the studies utilized deep learning models on dermoscopic images, demonstrating either the equal or superior performance of AI-based algorithms in detecting melanoma when compared to existing methods. Comparative studies of algorithm performance on dermoscopy images versus dermatologists revealed a higher ROC (>80%) for AI-based algorithms, with mean sensitivity of 83.01% and mean specificity of 85.58%. While the use of AI in clinical OCT for malignant melanoma diagnosis is still in its infancy, studies evaluating the use of AI on RCM images of melanoma have shown promising results in improving diagnosis accuracy and efficiency, allowing for the potential reduction of unnecessary biopsies. Studies evaluating machine learning in conjunction with OCT yielded accuracy of 95%, while studies evaluating RCM demonstrated a mean accuracy rate of 82.72%. The integration of AI into clinical platforms can have a significant impact on areas with disparities in melanoma incidence and higher mortality rates, such as rural communities or developing countries. However, ethical, legal, and patient privacy issues related to the use of AI must be considered before clinical integration. To date, this is the first review to synthesize the outcomes of the AI-trained analysis of melanoma detection using non-invasive imaging modalities. The information synthesized herein will aid in the future generation of AI-based diagnostic tools for melanoma while allowing for algorithm optimization. Further research should expand the datasets to include images from all skin tones and ethnicities, to develop more powerful, reliable, and clinically applicable algorithms. Our findings underscore the importance of continued efforts in the development and evaluation of AI-based techniques for the early detection of melanoma.

## Figures and Tables

**Table 1:** Review of AI-based techniques for dermoscopy image analysis.

Paper Title	Authors	Objective	AI Technique	Dataset	Diagnostic Accuracy Rate
Computer Aided Diagnosis of Melanoma Using Deep Neural Networks and Game Theory: Application on Dermoscopic Images of Skin Lesions	Foahom Gouabou et al. [12]	Development of a novel deep learning ensemble method for computer aided diagnosis of melanoma	Deep Learning Ensemble Method	ISIC 2018	AUROC = 0.93 for melanoma, BACC = 86%
Results of the 2016 International Skin Imaging Collaboration International Symposium on Biomedical Imaging challenge: Comparison of the accuracy of computer algorithms to dermatologists for the diagnosis of melanoma from dermoscopic images	Marchetti et al. [13]	Comparison of melanoma diagnostic accuracy of computer algorithms to dermatologists using dermoscopic images	Deep Learning	ISIC 2016	ROC of the top fusion algorithm = 0.86, mean ROC of dermatologists = 0.71. (*p* = 0.001)
Computer algorithms show potential for improving dermatologists’ accuracy to diagnose cutaneous melanoma: Results of the International Skin Imaging Collaboration 2017	Marchetti et al. [14]	Potential for improving dermatologists’ accuracy to diagnose cutaneous melanoma	Deep Learning	ISIC 2017	ROC of top-ranked computer algorithm = 0.87, ROC of dermatologists and residents = 0.66 (*p* < 0.001), dermatologists overall sensitivity = 76.0%, algorithm had superior specificity (85.0% vs. 72.6%, *p* = 0.001)
Lesion identification and malignancy prediction from clinical dermatological images	Xia et al. [15]	Two-stage approach that identifies all lesions in an image, estimates their likelihood of malignancy, and generates an image-level likelihood for high-level screening	Deep Learning	ISIC 2018	AUC of 0.959 based on dermoscopic images, which was augmented to an AUC of 0.961 using ISIC2018
An improved transformer network for skin cancer classification	Xin et al. [16]	Establishment of an improved transformer network named SkinTrans	Vision Transformer (VIT)	HAM10000	Accuracy = 94.3%
Skin Cancer Diagnosis Based on Neutrosophic Features with a Deep Neural Network	Singh et al. [17]	Computer-aided diagnosis system for the classification of a malignant lesion, where the acquired image is primarily pre-processed using novel methods	Deep Learning	PH2, ISIC 2017, ISIC 2018, and ISIC 2019	PH2 = 99.50%, ISIC 2017 = 99.33%, ISIC 2018 = 98.56%, and ISIC 2019 = 98.04%
SCDNet: A Deep Learning-Based Framework for the Multiclassification of Skin Cancer Using Dermoscopy Images	Naeem et al. [18]	Novel framework for the multiclassification of skin cancer types such as melanoma, melanocytic nevi, basal cell carcinoma, and benign keratosis	Deep Learning	ISIC 2019	Accuracy = 92.18%
Cancer-Net SCa: tailored deep neural network designs for detection of skin cancer from dermoscopy images	Lee et al. [19]	Deep neural network designs tailored for melanoma detection from dermoscopy images	Deep Learning	ISIC 2018	Sensitivity of 92.8%, PPV of 78.5%, and NPV of 91.2%
On the Automatic Detection and Classification of Skin Cancer Using Deep Transfer Learning	Fraiwan et al. [20]	Applicability of raw deep transfer learning in classifying images of skin lesions into seven possible categories	Deep Learning	HAM10000	71% sensitivity (i.e., recall) but 43.1% precision. Highest reported accuracy rate = 76.7%
Computational Intelligence-Based Melanoma Detection and Classification Using Dermoscopic Images	Vaiyapuri et al. [21]	Develops a novel computational intelligence-based melanoma detection and classification technique using dermoscopic images (CIMDC-DIs)	Computational Intelligence (CI) and Deep Learning (DL)	ISIC 2016, 2017, 2020	Maximum accuracy of 97.50%
Efficacy of a Deep Learning Convolutional Neural Network System for Melanoma Diagnosis in a Hospital Population	Martin-Gonzalez et al. [22]	Deep learning-based tool to differentiate between benign skin lesions versus melanoma in the hospital setting	Deep Learning	232 dermoscopic images	AUC = 0.813, sensitivity of 0.691, specificity of 0.802, and accuracy of 0.776
Deep Learning-Based Classification for Melanoma Detection Using XceptionNet	Lu et al. [23]	Automatic method for diagnosis of skin cancer from dermoscopy images	Deep Learning	HAM10000	Accuracy rate of 100% for the detection of melanoma, sensitivity rate of 94.05%, and precision rate of 97.07%
Melanoma Classification Using a Novel Deep Convolutional Neural Network with Dermoscopic Images	Kaur et al. [24]	Proposed a lightweight and less complex DCNN than other state-of-the-art methods to classify melanoma skin cancer with high efficiency	Deep Learning	ISIC 2016, ISIC 2017, and ISIC 2020	ISIC 2016 = 81.41%, ISIC 2017 = 88.23%, and ISIC 2020 = 90.42%
A Computer-Aided Diagnosis System Using Deep Learning for Multiclass Skin Lesion Classification	Arshad et al. [25]	Series-based new automated framework for multiclass skin lesion classification	Deep Learning	HAM10000	Accuracy = 91.7%
ZooME: Efficient Melanoma Detection Using Zoom-in Attention and Metadata Embedding Deep Neural Network	Xing et al. [26]	Zoom-in Attention and Metadata Embedding (ZooME) melanoma detection network	Deep Learning	ISIC 2020	92.23% AUC score, 84.59% accuracy, 85.95% sensitivity, and 84.63% specificity
AI outperformed every dermatologist in dermoscopic melanoma diagnosis, using an optimized deep-CNN architecture with custom mini-batch logic and loss function	Pham et al. [27]	Method for improving melanoma prediction on an imbalanced dataset by reconstructed appropriate CNN architecture and optimized algorithms	Deep Learning	17,302 dermoscopic images	AUC of 94.4%, sensitivity of 85.0%, and specificity of 95.0%
Computer-Aided Diagnosis Algorithm for Classification of Malignant Melanoma Using Deep Neural Networks	Kim et al. [28]	Tumor lesion segmentation model and a classification model of malignant melanoma	Deep Learning	ISIC 2017	Classification accuracy of malignant melanoma reached 80.06%
Skin cancer detection from dermoscopic images using deep learning and fuzzy k-means clustering	Nawaz et al. [29]	Fully automated method for segmenting skin melanoma at its earliest stage by employing a deep learning-based approach, namely faster region-based convolutional neural networks (RCNN) along with fuzzy k-means clustering (FKM)	DL-based approach	ISIC 2016, ISIC 2017, and PH2	ISIC 2016 = 95.40, ISIC 2017 = 93.1, and PH2 = 95.6%
A novel melanoma prediction model for imbalanced data using optimized SqueezeNet by bald eagle search optimization	Sayed et al. [30]	New model for the classification of skin lesions as either normal or melanoma	Deep Learning	ISIC 2020	Accuracy of 98.37%, specificity of 96.47%, sensitivity of 100%, f-score of 98.40%, and area under the curve of 99%
Ensemble Method of Convolutional Neural Networks with Directed Acyclic Graph Using Dermoscopic Images: Melanoma Detection Application	Foahom Gouabou et al. [31]	Novel ensemble scheme of convolutional neural networks (CNNs), inspired by decomposition and ensemble methods, to improve the performance of the CAD system	CAD/CNN	ISIC 2018	Best balanced accuracy = (76.6%)
Developing a Recognition System for Diagnosing Melanoma Skin Lesions Using Artificial Intelligence Algorithms	Alsaade et al. [32]	Development of feature-based and deep learning-based systems for melanoma classification	Deep learning and traditional artificial intelligence machine learning algorithms	PH2, ISIC 2018	Accuracy = PH2 (97.50%) and ISIC 2018 (98.35%)
Automated multi-class classification of skin lesions through deep convolutional neural network with dermoscopic images	Iqbal et al. [33]	Develop, implement, and calibrate an advanced deep learning model in the context of automated multiclass classification of skin lesions	Deep Convolutional Neural Network (DCNN) Model	ISIC-2017, ISIC-2018, and ISIC-2019	AUROC = 0.964, 94% precision, 93% sensitivity, and 91% specificity in ISIC-17
Melanoma diagnosis using deep learning techniques on dermatoscopic images	Jojoa Acosta et al. [34]	Two-stage DL-based method	Deep Learning	ISIC 2017	Overall accuracy (0.904), sensitivity (0.820), and specificity (0.925)
A new deep learning approach integrated with clinical data for the dermoscopic differentiation of early melanomas from atypical nevi	Tognetti et al. [35]	Deep convolutional neural network (DCNN) model able to support dermatologists in the classification and management of atypical melanocytic skin lesions (aMSL)	Deep Convolutional Neural Network (DCNN)	630 dermoscopic images	AUC = 90.3%, SE = 86.5%, and SP = 73.6%
Deep learning-level melanoma detection by interpretable machine learning and imaging biomarker cues	Gareau et al. [36]	Transparent machine learning technology (i.e., not deep learning) to discriminate melanomas from nevi in dermoscopy images and an interface for sensory cue integration	Transparent Machine Learning	349 images	AUROC was 0.71 ± 0.07, Sens = 98%, Spec = 36%
Effective Melanoma Recognition Using Deep Convolutional Neural Network with Covariance Discriminant Loss	Guo et al. [37]	Melanoma recognition method using deep convolutional neural network with covariance discriminant loss in dermoscopy images	Deep Learning	ISIC 2018	Sensitivity = 0.942 and 0.917
Deep Convolutional Neural Network for Melanoma Detection using Dermoscopy Images	R K et al. [38]	Propose a deep convolutional neural network for automated melanoma detection that is scalable to accommodate a variety of hardware and software constraints	Deep Learning	2150 dermosocpic images	Accuracy = 82.95%, sensitivity = 82.99%, and specificity = 83.89%
Dermoscopic diagnostic performance of Japanese dermatologists for skin tumors differs by patient origin: A deep learning convolutional nmeural network closes the gap	Minagawa et al. [39]	Compared the performance of 30 Japanese dermatologists with that of a DNN for the dermoscopic diagnosis of International Skin Imaging Collaboration (ISIC) and Shinshu (Japanese only) datasets to classify malignant melanoma, melanocytic nevus, basal cell carcinoma, and benign keratosis on non-volar skin	Deep Learning	ISIC and Shinsu (Japanese) dataset	Specificity of the DNN at the dermatologists’ mean sensitivity value = 0.962; Shinshu set = 1.00
DePicT Melanoma Deep-CLASS: a deep convolutional neural networks approach to classify skin lesion images	Nasiri et al. [40]	Approach to classify skin lesions using deep learning for early detection of melanoma in a case-based reasoning (CBR) system	Deep Learning	ISIC Archive	Accuracy = 0.77
Melanoma recognition by a deep learning convolutional neural network—Performance in different melanoma subtypes and localisations	Winkler et al. [41]	Investigated the diagnostic performance of a CNN with approval for the European market across different melanoma localizations and subtypes	Deep Learning	6 dermoscopic image sets (each set included 30 melanomas and 100 benign lesions of related localisations and morphology)	Sensitivities > 93.3%, specificities > 65%, receiver operating characteristic–area under the curve (ROC-AUC) > 0.926
Detection of Malignant Melanoma Using Artificial Intelligence: An Observational Study of Diagnostic Accuracy	Phillips et al. [42]	Evaluated the accuracy of an AI neural network (Deep Ensemble for Recognition of Melanoma (DERM)) to identify malignant melanoma from dermoscopic images	DERM	7102 dermoscopic images	ROC (AUC) of 0.93 (95% confidence interval: 0.92–0.94), and sensitivity and specificity of 85.0% and 85.3%
Deep neural networks are superior to dermatologists in melanoma image classification	Brinker et al. [43]	Automated dermoscopic melanoma image classification compared with dermatologists	Deep Learning	4204 biopsy-proven images of melanoma and nevi	Trained CNN achieved higher sensitivity of 82.3% (95% CI: 78.3–85.7%) and higher specificity of 77.9% (95% CI: 73.8–81.8%)
Deep learning outperformed 136 of 157 dermatologists in a head-to-head dermoscopic melanoma image classification task	Brinker et al. [44]	Performance of a deep learning algorithm trained by open-source images exclusively is compared to a large number of dermatologists covering all levels within the clinical hierarchy	Deep Learning	12,378 open-source dermoscopic images (training set), 100 images for comparison	At mean sensitivity of 74.1%, the CNN exhibited mean specificity of 86.5% (range 70.8–91.3%). At mean specificity of 60%, mean sensitivity of 87.5% (range 80–95%) was achieved by our algorithm
Deep Learning and Handcrafted Method Fusion: Higher Diagnostic Accuracy for Melanoma Dermoscopy Images	Hagerty et al. [45]	Approach that combines conventional image processing with deep learning by fusing the features from the individual techniques	Deep Learning	ISIC	AUC of 0.87, classification accuracy of 0.94
Man against machine: diagnostic performance of a deep learning convolutional neural network for dermoscopic melanoma recognition in comparison to 58 dermatologists	Haenssle et al. [46]	Compared a CNN’s diagnostic performance with a large international group of 58 dermatologists	Deep Learning	100 dermoscopic images	CNN ROC AUC was greater than the mean ROC area of dermatologists (0.86 versus 0.79, *p* < 0.01)
Skin Lesion Analysis towards Melanoma Detection Using Deep Learning Network	Li et al. [47]	Proposed two deep learning methods to address three main tasks emerging in the area of skin lesion image processing	Deep Learning	ISIC 2017	Highest accuracy = 0.912

**Table 2:** Review of AI-based techniques for optical coherence tomography image analysis.

Paper Title	Authors	Objective	AI Technique	Dataset	Diagnostic Accuracy Rate
Identification of Cancerous Skin Lesions Using Vibrational Optical Coherence Tomography (VOCT): Use of VOCT in Conjunction with Machine Learning to Diagnose Skin Cancer Remotely Using Telemedicine	Silver FH et al. [48]	Used VOCT and machine learning to evaluate the specificity and sensitivity of differentiating normal skin from skin cancers	Conventional Machine Learning	80 images	Sensitivity = 83.3%, specificity = 77.8%

**Table 3:** Review of AI-based techniques for reflectance confocal microscopy image analysis.

Paper Title	Authors	Objective	AI Technique	Dataset	Diagnostic Accuracy Rate
Convolutional Neural Network Approach to Classify Skin Lesions Using Reflectance Confocal Microscopy	Wodzinski et al. [49]	CNN-based approach to classify skin lesions using the reflectance confocal microscopy (RCM) mosaics	CNN	429 RCM mosaics	Test set classification accuracy = 87%
Semantic segmentation of reflectance confocal microscopy mosaics of pigmented lesions using weak labels	D’Alonzo et al. [50]	Development of a weakly supervised machine learning model to perform semantic segmentation of architectural patterns encountered in RCM mosaics	Deep Learning	157 RCM mosaics	Trained DNN achieved an average AUC of 0.969; Dice coefficient = 0.778

**Table 4:** Summary of publicly available datasets for dermoscopic images of melanoma.

Dataset	Total Dermoscopic Images
International Skin Imaging Collaboration (ISIC 2016)	1279
International Skin Imaging Collaboration (ISIC 2017)	2600
International Skin Imaging Collaboration (ISIC 2018)	11,527
International Skin Imaging Collaboration (ISIC 2019)	33,569
International Skin Imaging Collaboration (ISIC 2020)	44,108
PH2	200
Human Against Machine with 10,000 training images (HAM10000)	10,015
